# Comparative Nutritional, Rheological, and Lipid Profiling of Acorn Flours from Seven *Quercus* Species: A Scientific Framework for Functional Food Applications

**DOI:** 10.3390/foods15193398

**Published:** 2026-09-24

**Authors:** Leonardo G. Inácio, Daniela Godinho, Susana Bernardino, Clélia Afonso, Marko Jukić, Jasmina Lukinac, Raul Bernardino

**Affiliations:** 1 University of Leiria and Oeste, 2411-901 Leiria, Portugal; leonar-do.g.inacio@ulo.pt (L.G.I.); 4230139@my.ulo.pt (D.G.); susana.bernardino@ulo.pt (S.B.); clelia@ulo.pt (C.A.); 2 Marine and Environmental Sciences Centre (MARE), University of Leiria and Oeste, 2520-614 Peniche, Portugal; 3 Aquatic Research Network (ARNET), University of Leiria and Oeste, 2520-614 Peniche, Portugal; 4 Laboratory of Separation and Reaction Engineering-Laboratory of Catalysis and Materials (LSRE-LCM), University of Leiria and Oeste, 2411-901 Leiria, Portugal; 5 ALiCE—Associate Laboratory in Chemical Engineering, Faculty of Engineering, University of Porto, Rua Dr. Roberto Frias, 4200-465 Porto, Portugal; 6 Faculty of Food Technology Osijek, Josip Juraj Strossmayer University of Osijek, F. Kuhaca 18, 31000 Osijek, Croatia; marko.jukic@ptfos.hr

**Keywords:** acorn, *Quercus*, fatty acid, nutritional profile, starch, rheology, gluten-free, circular economy

## Abstract

Acorns are an underutilized natural resource with significant potential for sustainable food systems. This study evaluates the nutritional composition and lipid profile of acorn flours from seven *Quercus* species (*Q. pubescens*, *Q. ilex*, *Q. cerris*, *Q. petraea*, *Q. robur*, *Q. rotundifolia*, and *Q. suber*) collected in the Iberian Peninsula and Croatia across different harvest years. Proximate composition, including moisture, ash, protein, lipids, total dietary fiber, sugars, and carbohydrates, was determined using standard AOAC methods, and fatty acid profiles were analyzed by gas chromatography. Rheological properties measured with a micro-visco-amylograph (MVA) revealed significant interspecific differences in viscosity and paste stability, reflecting intrinsic structural variability in the starch fraction among the studied species. The results revealed notable variability among species, sample types, and collection conditions. Acorn flours were characterized by high carbohydrate content (up to 74.8% dw), moderate lipid levels (reaching 16.9% dw in *Q. pubescens* flour), and low protein content, while shells exhibited markedly higher dietary fiber values (up to 77.0% dw in *Q. robur* shell). Lipid profiles were dominated by unsaturated fatty acids, particularly oleic (up to 63.7%) and linoleic acids. The differences observed between samples reflect cumulative variability that may be associated with species, geographical origin, harvest year, and processing conditions, although these factors remain confounded in the current study. Because of the confounded sampling design, observed differences represent combined population diversity and should be validated in future balanced trial designs. These findings highlight the high nutritional density of acorns as sustainable, gluten-free ingredients and provide a scientific framework for their industrial use in developing novel functional foods and circular economy models.

## 1. Introduction

With the continuous growth of the global population and increasing environmental awareness, diversification of food sources has become a major challenge for global food security and sustainable nutrition [1,2]. One promising strategy involves the valorization of underutilized or neglected plant resources that were historically consumed but have gradually fallen into disuse. Among these, acorns represent a traditional food resource whose consumption almost disappeared during the twentieth century but has recently attracted renewed scientific and technological interest [3].

Acorns are the fruits of species belonging to the *Quercus* genus, which is widely distributed throughout the Northern Hemisphere, and has particular ecological and economic importance in Mediterranean ecosystems [4,5]. In this region, acorns are a key component of multifunctional agroforestry systems such as the Montado in Portugal and the Dehesa in Spain [6,7]. Oaks are well adapted to marginal environments, including nutrient-poor soils and prolonged drought, which makes them a resilient and sustainable source of biomass and fruit production [8]. In Portugal, *Quercus* species occupy approximately 36% of the national forest area, with cork oak (*Q. suber*) and holm oak (*Q. rotundifolia*) contributing to an estimated annual production of about 400 thousand tons of acorns [9,10]. Despite this abundance, it is estimated that around 55% of acorn production remains uncollected and is therefore underexploited [11]. Furthermore, while most research has focused on Iberian species, there is a lack of comparative data on populations from Southeast Europe, which are subject to different environmental pressures.

From a nutritional perspective, acorns are characterized by a high carbohydrate content, followed by moderate levels of lipids and relatively lower amounts of protein [12,13]. Carbohydrates typically represent between 78% and 91% of the acorn kernel, with starch accounting for approximately half of the kernel mass [5]. The lipid fraction varies significantly among species, generally ranging from 2% to 12% [14,15], while protein content typically ranges between 4% and 8% [13]. Moreover, the absence of gluten makes acorns particularly attractive as an alternative ingredient for gluten-free food formulations [16]. Nevertheless, previous studies have demonstrated considerable variability in the chemical composition of acorns depending on species, geographical origin and environmental conditions [2,10].

Despite the growing interest in acorns as a potential functional food ingredient, comparative studies evaluating the nutritional composition and bioactive properties of multiple *Quercus* species collected across different geographic regions remain limited. In particular, information regarding the influence of species, harvest year and collection site on the compositional variability of acorns is still scarce, which restricts the assessment of their technological and nutritional potential.

In this context, the present study aimed to characterize the nutritional composition and fatty acid profiles of acorn kernels and shells from seven *Quercus* species (*Q. pubescens*, *Q. ilex*, *Q. cerris*, *Q. petraea*, *Q. robur*, *Q. rotundifolia* and *Q. suber*) collected in the Iberian Peninsula and Croatia. Furthermore, to assess their technological potential for food formulations, the starch composition (total starch and amylose), hydration properties (water and oil absorption capacities), gelatinization and pasting behavior (using a micro-visco-amylograph), and Falling Number of the acorn flours were thoroughly evaluated. Due to biomass limitations for *Q. suber*, starch and rheological characterizations were restricted to six of the studied species. By distinguishing between flours (kernels) and shells, and analyzing samples across different harvest years, this work contributes to a better understanding of the nutritional potential of acorns. These findings provide a robust scientific basis for developing novel acorn-based food ingredients and support the transition toward more resilient and sustainable Mediterranean and Southern European oak ecosystems.

## 2. Materials and Methods

### 2.1. Sample Collection and Bioclimatic Context

Mature, healthy acorns from seven *Quercus* species were harvested from distinct bioclimatic regions in Croatia (Southeast Europe) and the Iberian Peninsula during peak physiological maturity (October–December) of each harvest season (2022–2024, depending on the sample; see Table 1). To ensure representativeness, three independent biological batches (1 kg each) were collected at each sampling location, with each batch consisting of pooled acorns from at least three individual trees.

Acorn samples from the Iberian Peninsula were partially provided by the company Landratech LDA (Azambuja, Portugal) and consisted of dehydrated kernels of *Q. robur*, *Q. suber*, and *Q. rotundifolia* collected in 2023. These kernels were subsequently milled in-house into flour prior to analysis. Additional *Q. rotundifolia* flour samples were obtained from Bragança, Portugal (2024) and Badajoz, Spain (2022). Unlike the remaining samples, the 2023 Iberian *Q. robur*, *Q. suber*, and *Q. rotundifolia* samples were received already dehydrated by the supplier and were therefore not subjected to the in-house drying protocol described in Section 2.2.

Samples from Croatia were collected across diverse habitats: *Q. pubescens* (Brzac/Glavotok, Island of Krk), *Q. ilex* (Glavotok, Island of Krk), *Q. cerris* (Borovik, Đakovo), *Q. petraea* (Mandićevac, Đakovo), and *Q. robur* (Slavir/Virovi, Otok). A summary of the sampling locations, year of collection, and sample coding used in this study is presented in Table 1. For each sampling site, three independent biological batches (1 kg each) were collected and pooled into a single uniform representative batch. Unless otherwise stated, all chemical and rheological measurements were performed in three analytical replicates (*n* = 3) obtained from these pooled batches, to ensure analytical precision.

Due to the limited amount of available biomass, the *Q. suber* sample was prioritized for core proximate analyses (moisture, ash, protein, lipids, total sugars) and fatty acid profiling, as these analyses were essential for the general nutritional characterization of all seven species. After a sufficient quantity of sample had been used for these analyses, the remaining amount of *Q. suber* material was insufficient for the additional, more demanding starch and rheological analyses, which require considerably larger amounts of flour (e.g., 15 g per replicate for the MVA analysis alone, plus further replicates for total starch, amylose, and WAC/OAC determination). For this reason, additional starch and rheological characterizations (starch composition, absorption capacities, and pasting properties) were performed on the remaining six species for which sufficient flour was available, whereas *Q. suber* was included in all other (proximate and lipid) analyses.

### 2.2. Sample Processing and Standardization

The preparation of acorn flours and milled shells followed the standardized protocol previously described by [17]. After collection, all acorns underwent rigorous visual inspection, and only mature, intact fruits free of pathogen damage or mechanical defects were retained for further processing. The kernels and shells were manually separated. The biomass was then dehydrated in a laboratory oven (B40, Memmert GmbH + Co. KG, Schwabach, Germany) at 40 °C for 24 h to ensure preservation and maintain the stability of the bioactive phenolic pool.

The acorn kernels (flour fraction) and shells were ground using an ultracentrifugal laboratory mill (ZM 200, Retsch GmbH, Haan, Germany) to obtain a fine powder with particles smaller than 200 μm. The resulting flours and milled shells were sealed in airtight containers and stored at room temperature, protected from light, until further chemical and lipid analyses. This standardized particle size was maintained across all species to ensure the comparability of extraction yields and nutritional data.

### 2.3. Derermination of Nutritional Profile

#### 2.3.1. Moisture and Ash

To determine moisture and ash content, the AOAC [18] protocol was used. Fresh samples were placed in an oven (Binder GmbH, Tuttlingen, Germany) at 105 °C for 2 h, and the weight was recorded after they reached room temperature. The dried samples were then placed in a muffle furnace at 525 °C for 5 h (Model B170, Nabertherm GmbH, Lilienthal, Germany). Once the weights were recorded, moisture content was calculated as a percentage of fresh weight (% fw) and ash content as a percentage of dry weight (% dw).

#### 2.3.2. Protein

Protein content was determined by quantifying nitrogen using the Kjeldahl method [18] with minor modifications. Dried biomass (0.8 g) was processed in a digester (Bloc Digest 6, JP SELECTA S.A., Barcelona, Spain) and in a semi-automatic distillation unit (UDK 139, VELP Scientifica Srl, Usmate Velate, Italy). The distillation output was titrated to determine the nitrogen content.

Protein content was calculated by multiplying the nitrogen value by a conversion factor of 6.25 and expressed as a percentage of dry weight (% dw).

#### 2.3.3. Lipids

Total lipid content was determined using the Folch method [19]. Briefly, a dried sample (1 g) was rehydrated with ultrapure water and homogenized in a chloroform–methanol solution (2:1, *v*/*v*) for 6 min. A 0.8% NaCl solution was then added, and the lipids were separated twice by centrifugation (4025 g, 10 min, 4 °C) (Model 5810R, Eppendorf SE, Horsholm, Denmark). The chloroform phase was transferred through a sodium sulfate column. The phase containing chloroform and lipids was evaporated using a rotary evaporator (Laborota 4000, Heidolph Instruments GmbH &amp; Co. KG, Schwabach, Germany). The extracted lipids were weighed and expressed as a percentage of dry weight (% dw).

#### 2.3.4. Determination of Total Dietary Fiber

Total dietary fiber (TDF) was quantified following the AOAC 985.29 method [18]. Briefly, the samples underwent enzymatic digestion with amylase, protease, and amyloglucosidase, followed by precipitation and washing of the residues with 78% and 95% ethanol and acetone. After drying, the residues were analyzed for protein and ash content using the previously described methods. Total dietary fiber content was calculated by subtracting the amounts of protein and ash from the final residue mass after washing.

#### 2.3.5. Total Sugars and Carbohydrates

Total sugar content was determined using the colorimetric Dubois’ method [20]. For sugar extraction, dry samples (5 mg) were hydrolyzed with sulfuric acid (H_2_SO_4_, 1 M) at 90 °C for 60 min (Precisterm 6000387, JP Selecta S.A., Barcelona, Spain). After hydrolysis, the liquid phase was separated by centrifugation (Eppendorf 5804, Horsholm, Denmark). Phenol (5%), concentrated H_2_SO_4_ (96%), and ultrapure water were then added, and the samples were allowed to cool to room temperature. Absorbance was measured at 485 nm using a UV–visible spectrophotometer (Evolution 201 UV-Vis spectrofotometer, Thermo Fisher Scientific, Waltham, MA, USA). Quantification of total sugars (total soluble carbohydrates) was performed using a glucose standard curve, and results were expressed as a percentage of dry weight (% dw).

Carbohydrate content was calculated by difference, subtracting the sum of ash, crude protein, total lipids, and TDF from 100% (on a dry weight basis), and expressed as % dw. In the specific case of *Q. suber*, where fiber was not separately quantified due to limited sample volume, the carbohydrate value represents the total carbohydrate fraction, including TDF.

### 2.4. Determination of Lipid Profile

The lipid profile of the acorn samples was determined by gas chromatography (GC) following the method described by Neves et al. [21]. Analyses were performed using a gas chromatograph (Finnigan Ultra Trace, Thermo Fisher Scientific, Waltham, MA, USA) equipped with a capillary column (60 m × 0.25 mm ID, 0.25 μm film thickness) (Thermo TR-FAME, Thermo Fisher Scientific, Waltham, MA, USA), an automatic injector (AS 3000, Thermo Fisher Scientific, Waltham, MA, USA), and a flame ionization detector (FID). Fatty acids were identified by comparison with the Supelco 37 FAME standard (Merck KGaA, Darmstadt, Germany), and results were expressed as the relative percentage of each fatty acid in relation to the total peak area (%TFA). The cumulative sums of saturated (ΣSFA), monounsaturated (ΣMUFA), and polyunsaturated (ΣPUFA) fatty acids were calculated by summing the relative percentages of all individually identified fatty acids belonging to each respective unsaturation class.

### 2.5. Total Starch Content

The total starch content of the acorn flour was determined using the Megazyme^®^ Total Starch Assay Procedure (K-TSTA; amyloglucosidase/α-amylase method; Megazyme Ltd., Bray, Ireland), in accordance with AACC International Method 76-13.01 [22]. Briefly, the sample was first pretreated to remove soluble sugars, followed by enzymatic hydrolysis of the starch. The starch was initially partially depolymerized using thermostable α-amylase, and then completely hydrolyzed to D-glucose with amyloglucosidase. The released D-glucose was quantitatively measured spectrophotometrically at 510 nm using a UV–Vis spectrophotometer (model UV-1280, Shimadzu Corporation, Kyoto, Japan) after reaction with the Glucose Determination Reagent (GOPOD). Total starch content was calculated from the absorbance values relative to the provided D-glucose standard and expressed as a percentage (%) on a dry weight basis. All analyses were performed in triplicate.

### 2.6. Amylose Content

The amylose and amylopectin contents of acorn starch were determined using the Megazyme^®^ Amylose/Amylopectin Assay Kit (K-AMYL; Megazyme Ltd., Bray, Ireland), based on the concanavalin A precipitation method described by [23], following the manufacturer’s instructions [24]. Briefly, the sample was completely dissolved by heating in dimethyl sulfoxide (DMSO). The starch was then precipitated with ethanol and recovered by centrifugation. To determine the amylose content, the amylopectin fraction was selectively precipitated by adding concanavalin A (Con A) and removed by centrifugation. The remaining amylose in the supernatant, as well as the total starch in a separate aliquot, were enzymatically hydrolyzed to D-glucose using α-amylase and amyloglucosidase. The released D-glucose was quantified spectrophotometrically at 510 nm using a UV–Vis spectrophotometer (model UV-1280, Shimadzu Corporation, Kyoto, Japan) after incubation with the Glucose Determination Reagent (GOPOD). The amylose content was expressed as a percentage of total starch, and the amylopectin content was calculated by subtracting the amylose percentage from 100%. All measurements were performed in triplicate.

### 2.7. Water and Oil Absorption Capacities (WAC and OAC)

The water absorption capacity (WAC) and oil absorption capacity (OAC) of acorn flour was determined using the centrifugal methods in a Multifuge 3L-R (Heraeus Instruments GmbH, Hanau, Germany) described by [25,26], respectively, with slight modifications. Briefly, 1.0 g of acorn flour was mixed with 10 mL of either distilled water (for WAC) or sunflower oil (for OAC) in a centrifuge tube. The mixture was vigorously shaken for 1 min and then allowed to stand at room temperature for 30 min to ensure complete hydration or solvation. The suspension was then centrifuged at 3000× *g* for 20 min. The supernatant was carefully decanted, and the tubes were inverted and drained for 30 min to remove excess unbound liquid. The weight of the remaining wet sediment was measured, and the capacities were calculated using the following equation: *WAC*/*OAC* (g/g) = (*W*_2_ − *W*_1_)/*W*_1_, where *W*_1_ is the initial weight of the dry acorn flour sample (g), and *W*_2_ is the weight of the wet sediment after centrifugation and draining (g). All measurements were performed in triplicate.

### 2.8. Pasting Properties and Falling Number (FN) Determination

The gelatinization and pasting behavior of the acorn flour samples were analyzed using a Micro Visco-Amylo-Graph (Brabender OGH, Duisburg, Germany). A slurry consisting of 15 g of flour (adjusted to a 14% moisture basis) and 100 mL of a 20 mM aqueous silver nitrate (AgNO_3_, Merck KGaA, Darmstadt, Germany) solution was prepared. The thermal profile began with heating from 30 to 92 °C at a constant rate of 5 °C/min, followed by an isothermal holding period at 92 °C for 5 min. The mixture was then cooled to 50 °C at 5 °C/min and maintained at this temperature for a final 1 min. A constant shear rate of 250 min^−1^ was applied throughout the entire operational cycle. The recorded parameters included pasting temperature, peak temperature, peak viscosity, breakdown viscosity, and setback viscosity. All viscosity values were reported in Brabender units (BU), and each sample was evaluated in duplicate.

Falling Number (FN) determination was performed on a Perten FN 1500 instrument (Perten Instruments AB, Stockholm, Sweden) according to AACC International Method 56-81.03 [27]. To inhibit potential endogenous α-amylase activity the method was modified by using a 20 mM aqueous silver nitrate (AgNO_3_) solution instead of demineralized water. A preliminary comparison between distilled water and the 20 mM AgNO_3_ solution showed no statistically significant differences in Falling Number (*p* > 0.05; Supplementary Table S1), indicating negligible endogenous α-amylase activity in the tested samples.

### 2.9. Statistical Analysis

All assays and analyses were performed in triplicate (*n* = 3) analytical (technical) replicates of this pooled master sample described in Section 2.1, except for dietary fiber determination, which was conducted in quadruplicate (*n* = 4). One-way analysis of variance (ANOVA) with Tukey’s Honestly Significant Difference (HSD) post hoc test was used to identify statistically significant differences between group means. Differences were considered statistically significant at *p* < 0.05. Results are presented as mean ± standard deviation. Pearson’s correlation coefficient (*r*) was used to assess the strength and direction of linear relationships among the measured starch properties. All statistical analyses were performed using IBM SPSS Statistics version 31.0 (IBM Corporation, New York, NY, USA).

## 3. Results and Discussion

### 3.1. Nutritional Profile

The comprehensive evaluation of the nutritional profiles of flours and shells from seven *Quercus* species reveals pronounced interspecific variability and provides a scientific basis for their potential industrial application. As shown in Table 2, acorns are a high-density energy resource composed primarily of carbohydrates and lipids, whereas shells are a substantial source of dietary fiber, supporting circular economy models through the valorization of food by-products. For standardized comparison, the following discussion focuses mainly on the flour/kernel fractions, as data distinguishing kernel and shell remain remarkably scarce in the current literature.

A clear limitation of the present study is that species, geographical origin, harvest year, and sample processing are confounded within the sampling matrix. Samples collected in different years and locations represent distinct populations distributed across a broad geographic range rather than a single population monitored over time. Consequently, inter-sample variation cannot be unequivocally attributed to specific environmental, climatic, or geographic drivers. These trends provide valuable baseline data for *Quercus* species but represent preliminary observations that require future confirmation using balanced, multifactorial sampling across identical locations and consecutive years.

#### 3.1.1. Moisture and Ash Content

The moisture content of the analyzed samples (6.24% to 10.70%) was intentionally standardized through controlled dehydration to ensure microbiological stability, prevent enzymatic degradation, and optimize milling precision [3]. These values are substantially lower than the moisture levels of fresh acorns, which typically range from 38% to 74% and are highly susceptible to rapid fungal proliferation. With the exception of the Iberian *Q. robur*, *Q. suber*, and *Q. rotundifolia* (2023) samples, which were received already dehydrated from the supplier (Landratech), all other samples underwent an in-house dehydration process (40 °C, 24 h) as described in Section 2.2. Despite this difference in drying origin, all samples reached similarly low final moisture contents suitable for standardized milling and analysis (<200 μm particle size), in line with established acorn flour preparation protocols [28]. The statistically significant differences observed in moisture content (Tukey HSD, *F*(14,44) = 45.758, *p* < 0.001) likely reflect intrinsic differences in the hygroscopic properties and water-binding capacity of the starch–protein matrix among species, as the molecular architecture of starch-rich matrices is known to govern water retention behavior [29]. When comparing samples of the same species for which both kernel and flour samples were available, flour generally had a significantly lower moisture content, except for *Quercus pubescens* and *Quercus cerris*. Among the *Q. rotundifolia* flour samples, the 2023 harvest exhibited higher moisture retention than the 2024 harvest (8.31% vs. 6.24% fw; Tukey HSD, *p* < 0.05). Significant intraspecific variation was also observed between the two *Q. robur* samples, with the Iberian sample exhibiting higher moisture content than the Croatian sample (7.68% vs. 7.38% fw; *p* < 0.05).

The moisture values obtained in the present study were significantly lower than those previously reported for non-dehydrated biomass, where moisture contents of approximately 38% were reported for *Q. cerris* and more than 42% for *Q. petraea* in Romanian populations [30]. Similarly, for *Q. ilex* and *Q. suber* collected in Spain, the literature reports moisture levels of 74% and 71%, respectively [31]. In contrast, the values obtained in the present study for *Q. rotundifolia* and *Q. robur* are more closely aligned with data reported for processed or air-dried acorns [28].

Regarding ash content, which represents the total inorganic mineral residue, values ranged from 1.24% dw (shell) for *Q. ilex* to 2.77% dw (flour) for *Q. cerris*, with significant interspecific variability confirmed by the Tukey HSD test (*F*(14,44) = 70.610, *p* < 0.001). In this study, no consistent difference was observed between ash content in shell and flour within the same species. However, notably for *Q. robur* flour, the sample collected in Croatia exhibited a significantly higher mineral density than the sample from the Iberian Peninsula (2.16% vs. 2.08% dw; Tukey HSD, *p* < 0.05). Similarly, interannual variation was observed in *Q. rotundifolia*, with the 2024 sample accumulating significantly higher ash content (2.15% dw) than the 2023 (1.54% dw) and 2022 (1.76% dw) harvests (*p* < 0.05).

The ash content obtained in this study was higher than that previously reported for *Q. robur* kernels [28], as well as that reported for *Q. cerris* and *Q. petraea* collected in Romania [30]. These discrepancies seem to indicate the influence of edaphic factors (soil composition) and regional environmental conditions on mineral accumulation in acorns. Moreover, our results for *Q. suber* and *Q. ilex* exceeded previously published values [31,32], whereas the mineral profile of *Q. rotundifolia* remained consistent with the ranges reported across different harvest years [4,28]. The values for *Q. pubescens* also closely matched reports on Mediterranean oak species [33].

#### 3.1.2. Protein Content

The protein content observed among the different species ranged from 2.13% in 7.00% the shell of *Quercus robur* to 7.00% in its kernel. Statistically significant differences were detected according to the Tukey HSD test (*F*(14,44) = 1917.890, *p* < 0.001). This range confirms that, although acorns are not primary protein sources, they have a high nutritional density that complements their carbohydrate-rich matrix, making them highly suitable for gluten-free diets [16,32].

Across all evaluated species, the flour/kernel fractions exhibited significantly higher protein contents than their corresponding shell fractions (Tukey HSD, *p* < 0.001), demonstrating distinct physiological nutrient compartmentalization. Among the *Q. rotundifolia* harvests, the 2022 sample had a slightly higher protein content (3.81% dw) than the 2023 and 2024 samples. Such interannual fluctuations are often attributed to varying environmental stressors and specific precipitation levels during peak physiological maturity [4,28].

These results are lower than previously reported values for *Q. rotundifolia* [4,28] and *Q. ilex* [31]. However, the value obtained in this study falls within the range reported for *Q. petraea* [30,34]. For *Q. suber*, *Q. cerris*, and *Q. pubescens*, the values obtained are higher than those previously reported in the literature [30,31,33]. For samples of the same species collected from different geographical locations, the values differ considerably, possibly indicating a relationship between edaphic factors and regional bioclimatic conditions and nitrogen accumulation in the fruit [2,30]. Specifically, the *Q. robur* sample from the Iberian Peninsula was the closest to the value reported by [28].

#### 3.1.3. Lipid Content

The lipid content ranged from 2.91% to 16.93% in *Q. suber* and *Q. pubescens* flour, respectively, with statistically significant differences among samples according to the Tukey HSD test (*F*(14,44) = 57.608, *p* < 0.001). The lipid content of *Q. suber* flour (2.91% dw) was significantly lower than that of all other evaluated flour samples (Tukey HSD, *p* < 0.001), identifying it as a distinct low-fat kernel fraction among Mediterranean oaks [32].

Although, as observed for protein content, flour samples tended to show higher lipid content, no statistically significant differences were found between shell and flour samples of the same species, with the exception of *Q. ilex*. This suggests that, unlike protein accumulation, lipogenesis in some *Quercus* species may occur more uniformly across different fruit tissues. Likewise, no statistically significant differences were observed among *Q. rotundifolia* samples collected in different years, nor between *Q. robur* samples collected from different geographical locations. This relative stability in lipid levels across harvest years suggests that lipid biosynthesis may be more genetically conserved than protein accumulation in these populations [4,35].

When comparing these results with previously published data, the lipid contents obtained in this study for *Q. pubescens*, *Q. petraea*, *Q. cerris*, *Q. ilex*, and *Q. robur* are higher than those previously reported [28,30,31,33,34]. For the different *Q. rotundifolia* samples, the values obtained are similar to those reported by [4,28]. In contrast, ref. [31] reported a lipid content for *Q. suber* acorns almost three times higher than the value obtained in the present study. These substantial discrepancies in the literature highlight the critical impact of sample processing, geographical origin, and the specific extraction methods used (e.g., Folch vs. Soxhlet) on total lipid recovery [28].

#### 3.1.4. Total Dietary Fiber

The total dietary fiber (TDF) content showed considerable variation among the samples, ranging from 9.61% in *Q. rotundifolia* flour (2023 harvest) to 77.00% in the shells of *Q. robur* from Croatia. This high variability, statistically confirmed by Tukey’s HSD test (*F*(14,44) = 45,330.627, *p* < 0.001), underscores the necessity of specific species and fruit-fraction selection for targeted functional food applications [3].

When comparing the different sample types, the shell samples had significantly higher total dietary fiber contents than the flour samples. This result was expected, as the shell is mainly composed of structural polysaccharides such as cellulose, hemicellulose, and lignin, similar to the shells of other nuts [36]. In addition, the standard deviation values were relatively high because the shell samples were difficult to mill and exhibited considerable heterogeneity. Since fiber is the main component, different proportions of pericarp and seed coat among replicates resulted in higher standard deviations. To minimize this variability in future studies, a more rigorous pre-homogenization step or specialized cryogenic milling may be required to achieve a more uniform particle distribution in the lignocellulosic matrix, thereby improving the reproducibility of TDF quantification in these recalcitrant tissues. Although extensive comparative data on acorn shells are currently lacking in the literature, the results align with studies on other agro-industrial by-products, such as hazelnuts, where TDF values exceeding 80% have been reported [36]. These findings suggest that acorn shells are a highly underexploited source of dietary fiber that could be valorized within a circular economy framework [3].

In the flour samples, differences were also observed, with the *Q. robur* flour sample from Portugal showing a significantly lower value than that collected in Croatia. This disparity likely reflects the influence of Southeast European environmental pressures and edaphic factors on the thickening of the fibrous matrix within the kernel [30]. Nevertheless, the values obtained are lower than the approximately 22.28% previously reported [28]. Likewise, *Q. rotundifolia* flour samples showed differences between the 2023 harvest and the other two harvest years. For this species, the values obtained are slightly below the previously reported 11.4% [4], whereas another study reports values between 17% and 22% dietary fiber [28,32]. For *Q. ilex*, the values obtained are slightly higher than those previously reported, which show considerable variability, ranging between 2.3% and 10.89% [31,32]. For the remaining species, no data on dietary fiber could be found; however, the values obtained fall within the typical range reported for acorns, which generally varies between 11% and 18% for most species [3,37,38].

This high dietary fiber content positions acorns as a highly promising functional ingredient, given the significant health benefits associated with these structural compounds [4]. Furthermore, the TDF in acorns consists predominantly of insoluble fiber fractions [28]. Similar fiber-rich acorn-based products have been associated with improved glycemic indices in patients with type 2 diabetes and with prebiotic effects through modulation of the gut–microbiota axis [30,31], suggesting that the isolated shell fiber fraction characterized here may hold comparable functional potential, although this remains to be confirmed for the specific fraction studied.

#### 3.1.5. Carbohydrates and Total Sugar Content

The carbohydrate (CHO) values presented in Table 2 include total sugars but exclude dietary fiber. These values range from 10.02% to 87.57% dw, corresponding to the shell of *Q. pubescens* and the flour of *Q. suber*, respectively. However, the carbohydrate value reported for the *Q. suber* sample includes dietary fiber, as there was insufficient material available for separate quantification. Although this inclusion provides an estimate of total carbohydrates, the absence of separate TDF data for *Q. suber* is a limitation that prevents a direct comparison of its available carbohydrate fraction with other species. Future analyses with larger sample volumes are needed to clarify whether the high CHO value (87.57%) is driven by available sugars or structural polysaccharides. When only available carbohydrate fractions are considered, the highest value is 74.81% for the *Q. rotundifolia* sample from 2024. Statistically significant differences were detected among samples according to Tukey’s HSD test (*F*(14,44) = 2106.841, *p* < 0.001).

The most pronounced compositional divergence was observed between the shell and flour fractions, reflecting their distinct biological roles. Shells are primarily composed of structural polysaccharides (fiber), whereas flour/kernel fractions are dominated by storage carbohydrates, specifically starch [13]. To the best of our knowledge, this study provides some of the first comparative data on carbohydrate content in acorn shells, as most existing literature focuses on the edible kernel only [13].

In the flour samples, carbohydrate content remained consistently high across species, generally ranging from 60% to 80% dw, which aligns with previous characterizations of acorns as a starch-rich plant resource [4,13].

Minor interannual fluctuations were noted for *Q. rotundifolia*, but the values (70% to 74.5% dw) are highly consistent with earlier reports for Mediterranean holm oak populations [4,28]. The values obtained for *Q. petraea*, *Q. ilex*, *Q. cerris*, and *Q. pubescens* were slightly lower than some previously reported data [30,31], whereas *Q. suber* values exceeded published figures, primarily due to inclusion of the TDF fraction, as previously discussed. For *Q. robur*, the results were well within the 59.17% to 76.30% range documented by other authors [28,34].

Total sugar content ranged from 3.90% to 17.64% dw, corresponding to the shell of *Q. pubescens* and the flour of *Q. robur*, respectively. Statistically significant variability among samples was detected using Tukey’s HSD test (*F*(14,44) = 61.786, *p* < 0.001). As with protein content, total sugar levels showed pronounced compartmentalization within the fruit: shell samples had significantly lower sugar concentrations than their respective flour/kernel fractions (Tukey HSD, *p* < 0.05), as observed for *Q. petraea* (9.91% vs. 4.12% dw), *Q. ilex* (10.08% vs. 4.97% dw), and *Q. robur* from Croatia (9.47% vs. 5.71% dw). The only exception was *Q. cerris*, whose flour and shell sugar contents did not differ significantly (7.76% vs. 4.88% dw), with both fractions among the lowest values recorded in this study. Among the flour/kernel fractions, sugar content varied significantly between *Q. rotundifolia* harvest years: the 2024 harvest had significantly lower sugar content (11.47% dw) than the 2022 and 2023 harvests (17.00% and 16.04% dw, respectively; Tukey HSD, *p* < 0.05), which did not differ significantly from each other. Similarly, the Iberian *Q. robur* sample exhibited significantly higher sugar content than its Croatian counterpart (17.64% vs. 9.47% dw; Tukey HSD, *p* < 0.05).

For total sugars, this study reveals higher concentrations than previously reported for *Q. ilex* and *Q. suber* [31], with *Q. suber* values more than twice as high as earlier findings. This elevated sugar profile, particularly in species such as *Q. robur* and *Q. rotundifolia*, suggests a natural sweetness that contributes to desirable flavor development and browning kinetics without requiring synthetic additives [34]. This potential was recently validated in gluten-free bakery formulations, where acorn flour incorporation improved consumer taste acceptance, aroma complexity, and crust color [17]. These results further emphasize that acorns from Southeast Europe and the Mediterranean are not only energy-rich but also offer unique sensory profiles driven by their soluble carbohydrate content [39].

As starch is the dominant carbohydrate fraction in acorn flours, its specific molecular organization and functional performance were further evaluated to assess its technological suitability for food systems.

### 3.2. Lipid Profile

The comprehensive fatty acid (FA) profiling of the seven *Quercus* species, as presented in Table 3, reveals a lipid composition dominated by highly unsaturated fatty acids, a key determinant of their nutritional and technological value. The analysis identified palmitic (C16:0), stearic (C18:0), oleic (C18:1 *n*-9 cis), linoleic (C18:2 *n*-6,9 cis), and α-linolenic (C18:3 *n*-3,6,9 cis; ALA) acids as the primary constituents, collectively accounting for approximately 95% of the total fatty acids (TFA). The reported PUFA, MUFA, and SFA contents represent the cumulative contribution of all fatty acids identified in each sample. The number of detected fatty acids varied across samples, ranging from 15 identified fatty acids in *Quercus rotundifolia* and *Quercus robur* flour samples collected in the Iberian Peninsula to 6 identified fatty acids in the shell sample of *Quercus pubescens* from Croatia.

Oleic acid was the predominant fatty acid in most samples, with concentrations ranging from 23.28% to 63.73% of TFA. Statistically significant differences among species and fractions were confirmed by Tukey’s HSD test (*F*(14,44) = 763.477, *p* < 0.001). The highest levels were recorded in *Q. rotundifolia* (reaching 63.73% in the 2024 harvest), followed by *Q. suber* (59.17%) and *Q. robur* from the Iberian Peninsula (57.39%). This high monounsaturated fatty acid (MUFA) content, combined with low saturated fat levels, yields a lipid profile remarkably similar to that of high-quality olive oil, reinforcing the potential of acorns for the production of functional edible oils [3,4].

Linoleic acid (C18:2 *n*-6,9 cis) was the second most abundant fatty acid, ranging from 16.47% to 39.04% of TFA, with significant differences confirmed by Tukey’s HSD test (*F*(14,44) = 435.096, *p* < 0.001). Our results show that Croatian species, particularly *Q. robur* (39.04%) and *Q. petraea* (36.94%), exhibit substantially higher linoleic acid levels than their Iberian counterparts. It remains to be determined whether this geographic divergence is strictly genetic or a plastic response to the specific bioclimatic conditions and precipitation patterns of the different harvest years (2022, 2023, and 2024). Variations in environmental temperature during fruit maturation are known to modulate the activity of fatty acid desaturases, potentially explaining the higher PUFA levels observed in Southeast European populations. This geographic divergence suggests that Southeast European environmental conditions may favor the accumulation of polyunsaturated fatty acids (PUFAs), which are essential for cardiovascular health and for reducing serum triglycerides [13,32].

A notable finding of this study is the high proportion of α-linolenic acid (ALA) in the shell samples, reaching 10.83% in *Q. pubescens* and 10.34% in *Q. petraea*. These values are significantly higher than those found in the corresponding flour fractions (*p* < 0.001, Tukey’s HSD), indicating specific compartmentalization of omega-3 fatty acids within the structural tissues of the fruit. The elevated ALA content in acorn shells suggests that this often-discarded by-product could be valorized as a source of essential *n*-3 fatty acids for the food and pharmaceutical industries [40].

Regarding saturated fatty acids (SFAs), palmitic acid (C16:0) was the most representative, with values ranging from 13.24% to 34.72% (*F*(14,44) = 499.007, *p* < 0.001). The shell samples, particularly those of *Q. pubescens*, *Q. petraea*, and *Q. robur*, showed a marked increase in palmitic acid compared with flours, leading to a compositional shift in which SFA became the dominant fraction (SFA > PUFA > MUFA), in contrast to the MUFA > PUFA > SFA pattern typically observed in the kernels [3]. Stearic acid (C18:0) levels remained relatively low (1.32% to 7.46%), with the highest values again observed in Croatian shell samples (*F*(14,44) = 187.260, *p* < 0.001).

Comparative analysis with the existing literature confirms the high quality of our samples. For *Q. rotundifolia*, our results align with findings from Mediterranean holm oak populations. However, for *Q. petraea*, our data reveal a higher PUFA-to-MUFA ratio than previously reported for Romanian populations, emphasizing the significant impact of geographical origin and bioclimatic pressures on the fatty acid signature of acorns [30].

In conclusion, the predominance of unsaturated fatty acids, together with the presence of bioactive omega-3 fractions in the shells, indicates that acorns from both the Iberian Peninsula and Southeast Europe are superior raw materials for the development of healthy, functional food ingredients [13,17].

### 3.3. Starch Composition and Functional Properties

#### 3.3.1. Starch Composition and Hydration-Related Properties

The starch composition and hydration-related functional properties of acorn flours obtained from six *Quercus* species are presented in Table 4. Significant interspecific differences (*p* < 0.05) were observed for all analyzed parameters, confirming that the botanical origin of acorns markedly influences both starch composition and functional behavior.

Total starch content ranged from 31.9% in *Q. cerris* to 47.8% in *Q. ilex*, with similarly high values for *Q. pubescens* (47.5%) and *Q. rotundifolia* (47.8%). Intermediate starch contents were found in *Q. robur* (42.9%) and *Q. petraea* (40.6%). This variability is consistent with previous reports showing that starch accumulation in acorns is highly species-dependent and reflects genetic differences in reserve deposition during seed development [29,41]. Although acorn starch is generally regarded as the dominant carbohydrate reserve, substantial quantitative variation among *Quercus* species has also been reported in studies on Mediterranean and Central European oaks [42].

In contrast to the pronounced variation in total starch, amylose content varied within a comparatively narrower interval, from 29.6% in *Q. robur* to 38.0% in *Q. rotundifolia*. Despite this relatively limited variability, differences in amylose proportion are technologically important because amylose plays a fundamental role in determining gelatinization behavior, paste stability and retrogradation during cooling [43,44]. The relatively high amylose contents observed in *Q. rotundifolia*, *Q. petraea* and *Q. ilex* therefore suggest a greater potential for developing stronger gel structures after gelatinization compared with species containing lower amylose levels.

The hydration behavior of acorn flours also differed significantly among species. Water absorption capacity (WAC) ranged from 0.71 g g^−1^ in *Q. robur* to 1.17 g g^−1^ in *Q. petraea*, while oil absorption capacity (OAC) varied between 0.59 g g^−1^ in *Q. rotundifolia* and 0.91 g g^−1^ in both *Q. robur* and *Q. pubescens*. These differences indicate that hydration and lipid-binding behavior are species-specific functional attributes that cannot be explained solely by starch concentration.

Water absorption primarily reflects the availability of hydrophilic functional groups capable of interacting with water molecules, together with the physical accessibility of starch granules and other flour constituents [45,46]. Although starch is the major water-binding component in acorn flour, water absorption is also influenced by the organization of the flour matrix, including damaged starch, cell wall polysaccharides, and other non-starch constituents [29]. This may explain why species with comparable starch contents showed different WAC values. For example, *Q. ilex* and *Q. rotundifolia* had almost identical total starch concentrations, yet *Q. ilex* showed higher WAC (0.99 vs. 0.89 g g^−1^). Even more pronounced was *Q. petraea*, which displayed the highest WAC despite having only intermediate starch content, suggesting that factors other than starch quantity contributed substantially to water binding.

Oil absorption capacity followed a different pattern. Unlike WAC, OAC showed no clear relationship with total starch content, indicating that lipid binding is governed predominantly by the surface characteristics of flour particles rather than starch concentration. Oil absorption has been associated with the availability of hydrophobic interaction sites, capillary retention within porous particles, and the exposure of nonpolar regions of proteins and other biopolymers [45,47]. Consequently, the relatively high OAC values observed for *Q. robur* and *Q. pubescens* likely reflect differences in flour microstructure rather than starch composition.

An important observation from Table 4 is that starch quantity alone does not fully explain the functional behavior of the investigated flours. Species with similar starch contents often differed in hydration capacity, whereas species with lower starch contents sometimes exhibited superior water absorption. This indicates that functional performance depends not only on the amount of starch but also on its molecular organization and its interactions with the surrounding flour matrix. Such behavior has been widely recognized in starch-rich botanical materials, where technological functionality results from the combined effects of starch composition, granule architecture, and interactions with non-starch components rather than starch concentration alone [29,44].

These compositional and hydration-related differences provide the basis for understanding the species-dependent variation in thermal and rheological behavior discussed in the following section. Because gelatinization and viscosity development are strongly influenced by both starch molecular structure and water availability, differences in WAC and starch composition are expected to be reflected in the pasting profiles obtained by micro-visco-amylograph analysis.

#### 3.3.2. Pasting Properties and Thermal Behavior

The pasting properties of acorn flours determined by micro-visco-amylograph (MVA) and falling number values are presented in Table 5. To evaluate the intrinsic physicochemical behavior of the starch fraction, both analyses were performed using an aqueous silver nitrate (AgNO_3_) solution to inhibit potential endogenous α-amylase activity. Preliminary analyses conducted in distilled water produced comparable results (Supplementary Table S1), indicating negligible endogenous amylolytic activity in the investigated acorn flours. Nevertheless, the use of AgNO_3_ ensured that the measured viscosity profiles and falling number values reflected the inherent properties of starch rather than possible enzymatic starch hydrolysis, allowing more reliable comparisons among *Quercus* species.

The onset of gelatinization differed significantly among species, with pasting temperatures ranging from 57.8 °C in *Q. pubescens* to 70.1 °C in *Q. robur*. Similarly, peak temperature varied between 73.8 °C (*Q. ilex*) and 83.2 °C (*Q. petraea*). Lower gelatinization temperatures generally indicate that starch granules require less thermal energy to initiate swelling, whereas higher temperatures suggest greater structural stability of the granular architecture [29,48]. The relatively low pasting temperatures observed for *Q. ilex* and *Q. pubescens* therefore indicate greater susceptibility of their starch granules to hydration and swelling during heating, while the higher temperatures recorded for *Q. robur*, *Q. rotundifolia,* and *Q. petraea* suggest more ordered granular organization.

Marked interspecific variation was observed in peak viscosity, which ranged from 379.6 BU in *Q. cerris* to 747.3 BU in *Q. pubescens*. High peak viscosity values were also recorded for *Q. ilex* (696.3 BU), whereas *Q. rotundifolia* exhibited only intermediate viscosity despite possessing one of the highest total starch contents. These observations indicate that starch concentration alone does not determine maximum paste viscosity. Instead, peak viscosity reflects the combined influence of starch content, granule swelling capacity, molecular architecture of amylopectin, and the physical constraints imposed by the surrounding flour matrix [44,46]. The relatively low peak viscosity of *Q. cerris* is consistent with its considerably lower starch content, whereas the behavior of *Q. rotundifolia* suggests that its starch granules may exhibit restricted swelling despite their abundance.

Final viscosity followed a pattern similar to peak viscosity, varying from 455.5 BU in *Q. cerris* to 859.1 BU in *Q. pubescens*. Because final viscosity reflects the viscosity of the starch paste after cooling, it represents the combined effects of granule integrity, molecular reassociation, and gel network formation [29,49]. The exceptionally high final viscosities observed for *Q. pubescens* and *Q. ilex* indicate the formation of relatively strong paste structures, whereas the substantially lower value recorded for *Q. cerris* suggests a less cohesive starch network.

Breakdown viscosity, representing the difference between peak viscosity and minimum viscosity during holding at high temperature, varied almost fourfold among species. The lowest values were observed in *Q. cerris* (57.9 BU) and *Q. rotundifolia* (60.9 BU), whereas *Q. pubescens* exhibited the highest breakdown (228.8 BU), followed by *Q. robur* (195.8 BU). A high breakdown value generally indicates extensive granule swelling accompanied by reduced resistance to mechanical and thermal stress, while lower values suggest greater stability of swollen starch granules during heating [46]. Consequently, although *Q. pubescens* developed the highest viscosity, its starch paste also appeared to be the least stable under continued heating, whereas *Q. cerris* and *Q. rotundifolia* maintained considerably greater structural integrity.

Setback viscosity, which reflects molecular reassociation during cooling and is commonly used as an indicator of retrogradation tendency, ranged from 143.9 BU in *Q. cerris* to 388.6 BU in *Q. pubescens*. Species exhibiting higher setback values generally also possessed higher final viscosities, indicating more extensive development of gel structure during cooling. Although amylose is recognized as the principal contributor to short-term retrogradation, the magnitude of setback is also influenced by amylopectin chain-length distribution, starch concentration, and interactions among starch granules within the paste [49,50]. This likely explains why setback did not perfectly follow amylose content alone. For example, *Q. rotundifolia* contained the highest amylose concentration but exhibited only intermediate setback, whereas *Q. pubescens*, with a lower amylose content, produced the highest setback value, suggesting that additional structural characteristics of starch contributed to gel formation.

The falling number values exhibited substantial variability, ranging from 97.9 s in *Q. cerris* to 533.5 s in *Q. rotundifolia*. Under conventional conditions, falling number is primarily interpreted as an indirect measure of α-amylase activity. However, because endogenous amylases were inhibited by AgNO_3_ in the present study, differences in falling number predominantly reflected the intrinsic flow behavior and thermal stability of gelatinized starch suspensions rather than enzymatic starch degradation. Consequently, the remarkably high falling number recorded for *Q. rotundifolia* indicates the formation of a highly resistant starch paste characterized by low fluidity under the standardized measurement conditions. Conversely, the low falling number of *Q. cerris* is consistent with its low starch content and limited viscosity development observed during MVA analysis.

An important observation emerging from the pasting profiles is that species exhibiting the highest starch content did not necessarily develop the highest viscosity or the greatest thermal stability. Instead, the results indicate that starch functionality is governed by the combined effects of starch quantity, amylose–amylopectin organization and granule architecture. This agrees with the current understanding that starch behaves as a hierarchical biological material, whose rheological properties cannot be predicted solely from compositional parameters [29,43]. Consequently, MVA analysis provided functional information that substantially extends beyond the compositional characteristics presented in Table 4 and offers valuable insight into the technological behavior of acorn flours during thermal processing.

From a functional food perspective, species such as *Q. pubescens* and *Q. ilex* may be suitable for formulating puddings, creams, or gels because of their higher peak and final viscosities. The practical applicability of *Q. pubescens* in gluten-free systems has already been confirmed, as we previously optimized and sensorially evaluated functional gluten-free cookies incorporating this flour [17]. Likewise, the greater thermal stability and more ordered granular organization of *Q. rotundifolia* indicate its potential use in gluten-free bakery products, where maintaining structural integrity during baking is critical. This application goes beyond mere hypothesis, as we have already demonstrated the technological viability of *Q. rotundifolia* flour by successfully developing and optimizing a functional gluten-free bread in an earlier study [51]. This technological potential is further supported by the low tannin content characteristic of *Q. rotundifolia* [52], which enhances its suitability for direct bakery applications.

#### 3.3.3. Correlation Analysis of Flour Functionality

The relationships among starch composition, hydration-related functional properties and pasting behavior are summarized in the Pearson correlation matrix in Figure 1. Because the analysis was performed using species mean values (*n* = 6), the correlation coefficients should be interpreted as indicators of the strength and direction of biological associations among *Quercus* species rather than as evidence of statistical dependence.

Among the compositional variables, total starch content showed the strongest positive associations with viscosity-related parameters, particularly final viscosity (*r* ≈ 0.86), setback viscosity (*r* ≈ 0.85), falling number (*r* ≈ 0.75) and peak viscosity (*r* ≈ 0.73). These relationships indicate that species containing larger amounts of starch generally developed more viscous and structurally stable pastes during heating and subsequent cooling. This observation agrees with the well-established role of starch concentration in determining paste consistency and gel strength, although starch quantity alone cannot fully explain rheological behavior because starch molecular organization also contributes substantially to viscosity development [29,43].

Amylose content displayed a distinctly different pattern. The strongest association was observed with falling number (*r* ≈ 0.81), whereas only weak relationships were found with peak viscosity, final viscosity, and setback viscosity. Although amylose is widely recognized as an important determinant of retrogradation, the relatively modest correlation between amylose content and setback suggests that retrogradation behavior in acorn flour cannot be attributed solely to amylose concentration. Instead, the results support previous findings indicating that amylopectin architecture, molecular interactions and starch granule structure substantially influence paste reorganization during cooling [44,49,50]. This pattern is illustrated by *Q. rotundifolia*, which had the highest amylose content but did not exhibit the highest setback value, whereas *Q. pubescens* developed the greatest setback despite a lower amylose proportion.

The strongest relationships in the dataset were observed among the rheological parameters determined by micro-visco-amylograph. Peak viscosity, final viscosity and setback viscosity were all strongly positively associated with one another (*r* ≈ 0.91–0.98), showing that starch systems producing extensive swelling during heating also generated stronger gel structures upon cooling. Likewise, peak viscosity showed a strong positive association with breakdown viscosity (*r* ≈ 0.90), indicating that starches capable of extensive granule swelling also tended to be more susceptible to structural disintegration under prolonged thermal and mechanical stress. Such behavior is characteristic of starch systems in which high swelling capacity is accompanied by reduced granule stability [46].

Thermal characteristics exhibited the opposite tendency. Both pasting temperature and peak temperature were negatively associated with viscosity-related parameters, particularly peak viscosity (*r* ≈ −0.71) and setback (*r* ≈ −0.70). This inverse relationship suggests that starches initiating gelatinization at lower temperatures generally developed greater viscosity during heating. Similar relationships have been described for starches from numerous botanical sources and are commonly attributed to differences in crystalline organization and granule architecture, which determine the thermal energy required for granule swelling [29,48].

The interpretation of falling number deserves particular consideration because measurements were performed in the presence of AgNO_3_ to suppress endogenous α-amylase activity. Under these conditions, differences in falling number predominantly reflect the intrinsic rheological behavior of gelatinized starch rather than enzymatic starch degradation. The positive associations of falling number with total starch, amylose and final viscosity therefore indicate that samples exhibiting greater resistance to stirrer penetration also formed more cohesive starch networks during cooling. The close relationship between falling number and starch compositional characteristics supports the use of this parameter as an additional indicator of starch functional integrity when enzymatic interference is eliminated.

Hydration-related properties showed comparatively weaker associations with starch functionality. Water absorption capacity displayed moderate positive relationships with amylose, final viscosity, and falling number, suggesting that increased water binding may facilitate viscosity development by improving starch hydration before gelatinization. However, the relatively weak association between WAC and total starch demonstrates that water absorption is governed by the combined effects of starch, cell wall polysaccharides, damaged starch, and flour microstructure rather than starch concentration alone [29,46]. Oil absorption capacity, in contrast, exhibited no consistent relationship with viscosity parameters and was negatively associated with amylose and falling number. This behavior indicates that lipid retention is controlled primarily by surface characteristics of flour particles and the exposure of hydrophobic regions rather than by the physicochemical properties of starch itself [45].

Taken together, the correlation analysis supports a hierarchical structure–function model for acorn flours. Total starch content represents the principal compositional determinant of viscosity development, whereas amylose appears to influence specific aspects of starch organization, particularly those associated with paste stability rather than maximum viscosity. Water absorption contributes to, but does not govern, starch functionality, while oil absorption represents a largely independent characteristic of the flour matrix. Most importantly, the exceptionally strong interrelationships among the MVA parameters confirm that the thermal behavior of acorn starch is internally coherent and largely reflects intrinsic physicochemical properties of the starch fraction. Because endogenous α-amylase activity was suppressed during analysis, these relationships can be attributed primarily to differences in starch organization among *Quercus* species rather than to enzymatic effects. Collectively, these findings showing that the technological functionality of acorn flours is determined by the interaction between starch composition and structural characteristics of starch, highlighting the importance of species selection when acorn flour is intended for food applications requiring specific rheological performance.

## 4. Conclusions

This study confirms that acorns from diverse *Quercus* species are high-density energy resources, dominated by carbohydrates (up to 74.8% dw) and featuring a fatty acid profile rich in oleic and linoleic acids, with a MUFA/PUFA/SFA ratio that closely resembles that of high-grade olive oil. While kernels and flours provide a balanced matrix with moderate lipid and protein contents, acorn shells emerge as a substantial, underexploited reservoir of dietary fiber (up to 77.0% dw), offering significant potential for circular economy models through by-product valorization. The observed variability in these profiles underscores the pronounced diversity among the studied acorn samples. However, because geographical origin, harvest year, and processing conditions are confounded in the current experimental design, further systematically controlled research is needed to determine the independent effects of these environmental and technological factors on the fruit’s nutritional architecture.

Our findings move from basic composition to technological application, showing that the functionality of acorn flour is governed by the complex interaction between starch composition and molecular architecture rather than by starch concentration alone. This relationship is central to species-specific industrial use: species such as *Q. pubescens* and *Q. ilex* may be suitable for gel-based formulations due to their superior final viscosity, whereas *Q. rotundifolia* shows promising thermal stability characteristics for gluten-free bakery systems.

In summary, acorns represent a valuable, sustainable, and underutilized nutritional resource, and this work provides a scientific framework for their functional food applications by linking baseline physicochemical data to specific technological uses. The technological suitability of *Q. pubescens* and *Q. rotundifolia* flours for thermally stable, gluten-free bakery products has been empirically supported by recent formulation and optimization studies, while the high paste viscosity of *Q. ilex* highlights its potential for gel-based formulations. Future research should build directly on these findings through pilot-scale processing trials, sensory evaluation of proposed product applications, and bioavailability studies of shell-derived fiber and lipid fractions to further validate their industrial potential.

## Figures and Tables

**Figure 1:**
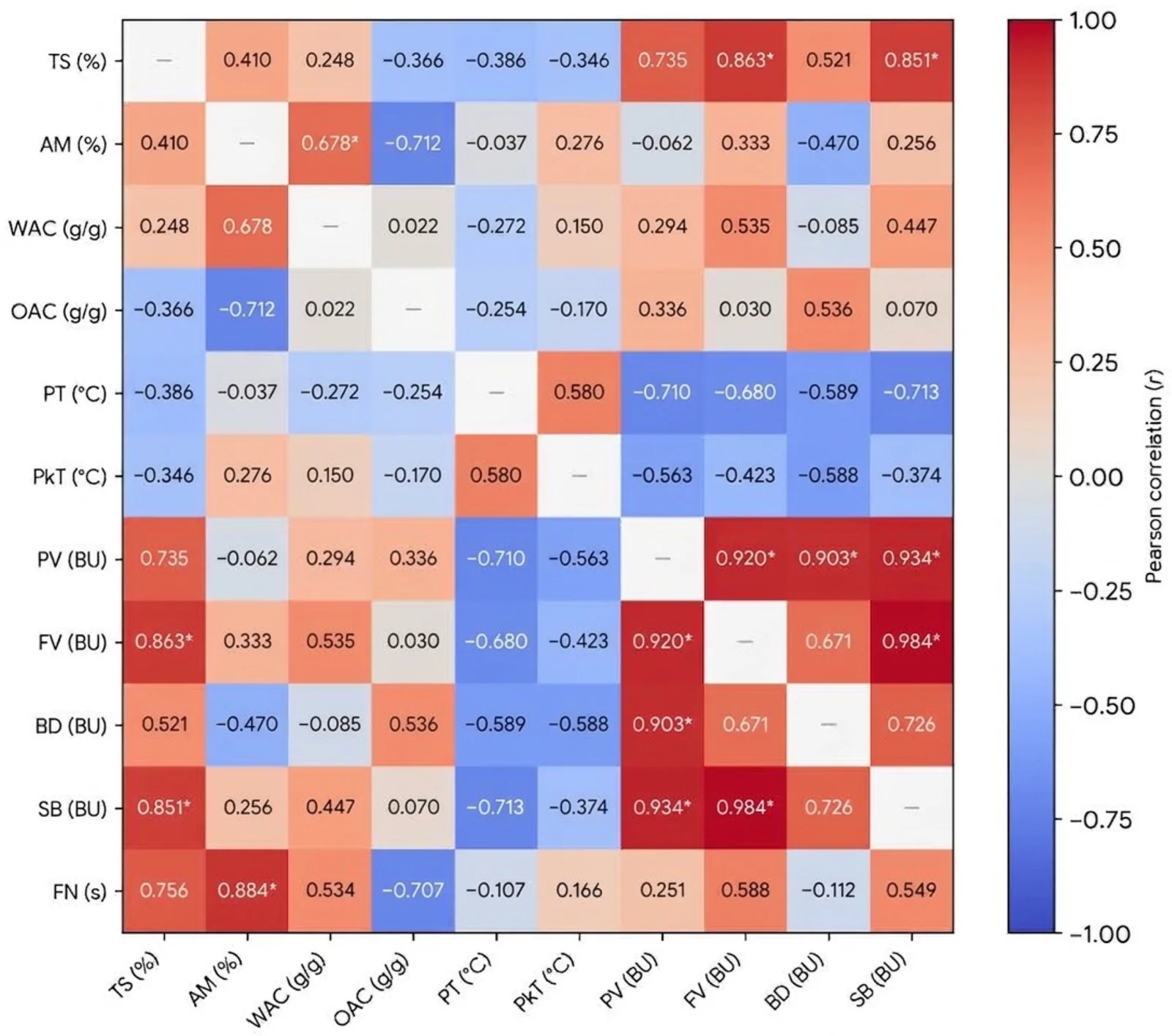
Pearson correlation coefficients among starch composition, absorption and pasting properties of acorn flours. * *p* < 0.05; TS, total starch; AM, amylose; PT, pasting temperature; PkT, peak temperature; PV, peak viscosity; FV, final viscosity; BD, breakdown viscosity; SB, setback viscosity; FN, falling number; WAC, water absorption capacity; OAC, oil absorption capacity.

**Table 1:** Characteristics of the studied *Quercus* species samples, including sample type, year of collection, geographical origin, and study codes.

Acorn Species	Sample Type	Year	Location	Sample Code
*Q. suber*	Whole kernel	2023	Iberian Peninsula	Qsub
*Q. robur*	Whole kernel	2023	Iberian Peninsula	Qrob
*Q. rotundifolia*	Whole kernel	2023	Iberian Peninsula	Qrot23
*Q. rotundifolia*	Flour	2022	Badajoz, Spain	Qrot22
*Q. rotundifolia*	Flour	2024	Bragança, Portugal	Qrot24
*Q. pubescens*	Flour	2024	Glavotok, Croatia	Qpub F
*Q. pubescens*	Milled Shell	2024	Glavotok, Croatia	Qpub S
*Q. ilex*	Flour	2024	Glavotok, Croatia	Qilex F
*Q. ilex*	Milled Shell	2024	Glavotok, Croatia	Qilex S
*Q. cerris*	Flour	2024	Borovik, Croatia	Qcer F
*Q. cerris*	Milled Shell	2024	Borovik, Croatia	Qcer S
*Q. petraea*	Flour	2024	Mandićevac, Croatia	Qpet F
*Q. petraea*	Milled Shell	2024	Mandićevac, Croatia	Qpet S
*Q. robur*	Flour	2024	Virovi, Croatia	Qrob F
*Q. robur*	Milled Shell	2024	Virovi, Croatia	Qrob S

F—flour (kernel) fraction; S—milled shell fraction. Samples from the Iberian Peninsula (2023) were provided as dehydrated kernels by Landratech, Portugal.

**Table 2:** Nutritional profile data for the different acorn species, expressed as % fresh weight (% fw for moisture) and % dry weight (% dw for the remaining parameters).

Sample Code	Moisture	Ash	Protein	Lipids	TDF	Total Sugar	CHO
Qsub	10.70 ± 0.40 ^e^	2.55 ± 0.12 ^gh^	6.98 ± 0.08 ^k^	2.91 ± 0.43 ^a^	n.d.	15.80 ± 0.84 ^ef^	87.57 ± 0.46 ^i^
Qrob	7.68 ± 0.15 ^bc^	2.08 ± 0.13 ^de^	7.00 ± 0.04 ^k^	7.06 ± 0.48 ^bcd^	12.66 ± 0.14 ^d^	17.64 ± 0.09 ^f^	71.26 ± 0.62 ^g^
Qrot23	8.31 ± 0.27 ^cd^	1.54 ± 0.08 ^b^	3.42 ± 0.07 ^c^	10.78 ± 0.70 ^fgh^	9.61 ± 0.28 ^a^	16.04 ± 0.73 ^f^	74.77 ± 0.98 ^h^
Qrot22	8.50 ± 0.59 ^d^	1.76 ± 0.03 ^bcd^	3.81 ± 0.06 ^ef^	12.00 ± 0.25 ^gh^	10.97 ± 0.45 ^c^	17.00 ± 1.00 ^f^	71.47 ± 0.83 ^g^
Qrot24	6.24 ± 0.02 ^a^	2.15 ± 0.04 ^ef^	3.63 ± 0.07 ^de^	10.20 ± 1.20 ^efg^	10.79 ± 0.16 ^c^	11.47 ± 0.46 ^d^	74.81 ± 1.22 ^h^
Qpub F	8.72 ± 0.07 ^d^	2.10 ± 0.01 ^ef^	6.26 ± 0.11 ^j^	16.93± 1.66 ^j^	9.80± 2.00 ^ab^	12.28 ± 1.11 ^de^	64.89 ± 1.74 ^f^
Qpub S	8.70 ± 0.2 ^d^	2.32 ± 0.18 ^fg^	2.72 ± 0.08 ^b^	15.65 ± 0.73 ^ij^	69.30 ± 13.90 ^i^	3.90 ± 0.39 ^a^	10.02 ± 0.56 ^a^
Qilex F	7.76 ± 0.13 ^bc^	1.90 ± 0.01 ^cde^	3.97 ± 0.07 ^fg^	13.72 ± 0.76 ^hi^	13.30 ± 2.70 ^de^	10.08 ± 1.02 ^cd^	67.10 ± 0.81 ^f^
Qilex S	8.66 ± 0.06 ^d^	1.24 ± 0.08 ^a^	2.85 ± 0.04 ^b^	8.37 ± 1.40 ^def^	68.80 ± 13.80 ^i^	4.97± 0.38 ^ab^	18.73 ± 1.34 ^c^
Qcer F	8.84 ± 0.13 ^d^	2.77 ± 0.17 ^h^	5.81 ± 0.06 ^i^	6.54 ± 1.20 ^bcd^	10.20 ± 2.00 ^b^	7.76 ± 0.60 ^bc^	74.66 ± 1.32 ^h^
Qcer S	9.78 ± 0.07 ^e^	2.68 ± 0.12 ^h^	3.58 ± 0.13 ^cd^	4.73 ± 0.24 ^ab^	66.40 ± 12.30 ^h^	4.88± 0.39 ^ab^	22.45 ± 0.14 ^d^
Qpet F	7.01 ± 0.16 ^ab^	2.75 ± 0.03 ^h^	5.31 ± 0.01 ^h^	4.89 ± 0.34 ^abc^	13.40 ± 2.70 ^e^	9.91 ± 0.95 ^cd^	73.64 ± 0.31 ^gh^
Qpet S	8.98 ± 0.22 ^d^	2.53 ± 0.12 ^gh^	2.74 ± 0.06 ^b^	4.54 ± 1.98 ^ab^	63.40 ± 12.70 ^g^	4.12 ± 0.30 ^a^	26.78 ± 1.94 ^e^
Qrob F	7.38 ± 0.09 ^b^	2.16 ± 0.06 ^ef^	4.14 ± 0.04 ^g^	7.82 ± 0.24 ^cde^	14.85 ± 0.17 ^f^	9.47 ± 0.45 ^cd^	71.02 ± 0.64 ^g^
Qrob S	8.99 ± 0.0 ^d^	1.61 ± 0.06 ^bc^	2.13 ± 0.06 ^a^	5.88 ± 0.67 ^bcd^	77.00 ± 15.40 ^j^	5.71 ± 0.56 ^ab^	13.36 ± 0.74 ^b^

Values are expressed as mean ± SD (*n* = 3, except for Total Dietary Fiber (TDF), where *n* = 4). F—flour (kernel) fraction; S—milled shell fraction; TDF—Total Dietary Fiber; CHO—Available Carbohydrates; n.d.—no data. Different lowercase letters (a–k) within the same column indicate statistically significant differences (Tukey HSD, *p* < 0.001). For *Q. suber* (Qsub), CHO values include TDF because it was not quantified separately.

**Table 3:** Lipid profile data for the different acorn species, expressed as % of total fatty acids (mean ± SD).

Sample Code	C16:0	C18:0	C18:1 *n*-9 cis	C18:2 *n*-6,9 cis	C18:3 *n*-3,6,9 cis ALA	ƩSFA	ƩMUFA	ƩPUFA
Qsub	13.88 ± 0.19 ^ab^	1.32 ± 0.05 ^a^	59.17 ± 0.15 ^f^	20.60 ± 0.12 ^cdef^	1.99 ± 0.01 ^b^	16.93 ± 0.25	59.72 ± 0.12	23.35 ± 0.13
Qrob	15.31 ± 0.39 ^bc^	1.36 ± 0.08 ^a^	57.39 ± 0.44 ^f^	21.61 ± 0.10 ^efg^	1.74 ± 0.01 ^ab^	17.90 ± 0.45	57.96 ± 0.45	24.14 ± 0.09
Qrot23	15.02 ± 0.18 ^bc^	3.08 ± 0.02 ^b^	62.34 ± 0.15 ^g^	16.47 ± 0.02 ^a^	0.72 ± 0.01 ^a^	17.14 ± 0.22	64.05 ± 0.19	18.81 ± 0.03
Qrot22	13.46 ± 0.24 ^a^	2.63 ± 0.02 ^b^	63.28 ± 0.17 ^g^	17.27 ± 0.02 ^a^	0.73 ± 0.01 ^a^	19.04 ± 0.14	62.92 ± 0.15	18.03 ± 0.03
Qrot24	13.24 ± 0.16 ^a^	2.69 ± 0.01 ^b^	63.73 ±0.14 ^g^	17.90 ± 0.03 ^ab^	0.72 ± 0.02 ^a^	16.91 ± 0.17	64.47 ± 0.15	18.62 ± 0.02
Qpub F	15.76 ± 0.44 ^cd^	1.66 ± 0.08 ^a^	50.21 ± 0.55 ^e^	29.55 ± 0.23 ^i^	2.08 ± 0.04 ^b^	17.91 ± 0.49	50.45 ± 0.58	31.64 ± 0.27
Qpub S	34.72 ± 0.28 ^j^	4.86 ± 0.15 ^d^	27.07 ± 0.76 ^b^	20.10 ± 0.32 ^cde^	10.83 ± 0.76 ^g^	42.01 ± 0.28	27.07 ± 0.76	30.92 ± 1.04
Qilex F	17.69 ± 0.22 ^ef^	3.19 ± 0.03 ^b^	57.54 ± 0.11 ^f^	19.60 ± 0.10 ^bcd^	1.37 ± 0.06 ^ab^	21.37 ± 0.14	57.66 ± 0.10	20.97 ± 0.09
Qilex S	23.58 ± 1.44 ^g^	4.37 ± 0.36 ^cd^	45.53 ± 2.74 ^d^	19.24 ± 0.48 ^bc^	4.85 ± 0.76 ^d^	30.38 ± 1.61	45.53 ± 2.74	24.09 ± 1.14
Qcer F	18.18 ± 0.08 ^ef^	1.51 ± 0.15 ^a^	50.33 ± 0.06 ^e^	27.37 ± 0.09 ^h^	2.04 ± 0.03 ^b^	20.05 ± 0.14	50.54 ± 0.08	29.41 ± 0.07
Qcer S	24.33 ± 0.56 ^gh^	2.68 ± 0.19 ^b^	40.26 ± 0.45 ^c^	22.15 ± 0.34 ^fg^	6.67 ± 0.24 ^e^	29.90 ± 0.85	41.27 ± 0.51	28.82 ± 0.35
Qpet F	19.01 ± 0.32 ^f^	1.50 ± 0.22 ^a^	38.07 ± 0.29 ^c^	36.94 ± 0.20 ^j^	3.49 ± 0.02 ^c^	21.13 ± 0.09	38.44 ± 0.28	40.43 ± 0.19
Qpet S	30.26 ± 0.57 ^i^	4.13 ± 0.35 ^c^	25.79 ± 0.74 ^ab^	21.10 ± 1.02 ^def^	10.34 ± 0.06 ^g^	41.81 ± 1.39	26.75 ± 0.71	31.44 ± 0.97
Qrob F	17.02 ± 0.17 ^de^	1.51 ± 0.31 ^a^	40.03 ± 1.22 ^c^	39.04 ± 1.18 ^k^	2.01 ± 0.24 ^b^	18.63 ± 0.13	40.32 ± 1.20	41.06 ± 1.32
Qrob S	25.52 ± 0.60 ^h^	7.46 ± 0.46 ^e^	23.28 ± 0.78 ^a^	23.12 ± 1.35 ^g^	8.55 ± 0.88 ^f^	44.26 ± 1.30	24.07 ± 0.74	31.67 ± 0.61

C16:0, palmitic acid; C18:0, stearic acid; C18:1 *n*-9 cis, oleic acid; C18:2 *n*-6,9 cis, linoleic acid; C18:3 *n*-3,6,9 cis (ALA), α-linolenic acid; SFA, saturated fatty acids; MUFA, monounsaturated fatty acids; PUFA, polyunsaturated fatty acids. ΣSFA, ΣMUFA, and ΣPUFA represent the cumulative sums of all individual fatty acids identified in each sample (ranging from 6 in shell samples up to 15 in whole flour samples). Lowercase letters (a–k) within the same column indicate statistically significant differences (Tukey HSD, *p* < 0.001).

**Table 4:** Starch composition and absorption properties of acorn flours.

Species	Total Starch (%)	Amylose (%)	WAC (g/g)	OAC (g/g)
*Q. cerris*	31.9 ± 0.4 ^c^	32 ± 2.3 ^bc^	0.78 ± 0.01 ^e^	0.86 ± 0.01 ^b^
*Q. ilex*	47.8 ± 0.6 ^a^	35 ± 2.2 ^ab^	0.99 ± 0.01 ^b^	0.81 ± 0.01 ^c^
*Q. petraea*	40.6 ± 0.2 ^b^	36 ± 1.9 ^ab^	1.17 ± 0.01 ^a^	0.89 ± 0.01 ^ab^
*Q. robur*	42.9 ± 0.3 ^b^	29.6 ± 1.4 ^c^	0.71 ± 0.01 ^f^	0.91 ± 0.02 ^a^
*Q. pubescens*	47.5 ± 1.5 ^a^	33.3 ± 0.4 ^bc^	0.94 ± 0.01 ^c^	0.91 ± 0.01 ^a^
*Q. rotundifolia*	47.8 ± 0.4 ^a^	38 ± 1.4 ^a^	0.89 ± 0.01 ^d^	0.59 ± 0.01 ^d^

Values are mean ± SD. Different letters in the same column indicate statistically significant difference between acorn flour samples according to Tukey HSD test (*p* < 0.05). All evaluated samples correspond exclusively to the acorn flour (kernel) fraction. *Q. pubescens*, *Q. ilex*, *Q. cerris*, *Q. petraea*, and *Q. robur* (2024) originate from Croatia, whereas *Q. rotundifolia* (2024) originates from Portugal. *Q. suber* was excluded from these analyses due to flour biomass constraints.

**Table 5:** Pasting properties of acorn flours.

Species	Pasting Temperature (°C)	Peak Temperature (°C)	Peak Viscosity (BU)	Final Viscosity (BU)	Breakdown Viscosity (BU)	Setback Viscosity (BU)	Falling Number (s)
*Q. cerris*	66.5 ± 1 ^b^	81 ± 0.4 ^a^	379.6 ± 4.6 ^e^	455.5 ± 3.9 ^d^	57.9 ± 6.6 ^c^	143.9 ± 11.6 ^d^	97.9 ± 5.7 ^d^
*Q. ilex*	59.5 ± 0.6 ^c^	73.8 ± 1.8 ^b^	696.3 ± 17.3 ^b^	839.2 ± 14.2 ^a^	175.8 ± 15.9 ^b^	335.7 ± 11.9 ^b^	346.2 ± 21.9 ^b^
*Q. petraea*	68 ± 0.4 ^ab^	83.2 ± 0.9 ^a^	559.4 ± 7.4 ^c^	716.3 ± 11.3 ^b^	96.9 ± 3.3 ^c^	271.7 ± 5.6 ^c^	349.2 ± 16.3 ^b^
*Q. robur*	70.1 ± 0.2 ^a^	78.8 ± 0.5 ^ab^	598.4 ± 9.1 ^c^	637.4 ± 7.4 ^c^	195.8 ± 13.1 ^ab^	244.8 ± 6.6 ^c^	160.8 ± 19.8 ^c^
*Q. pubescens*	57.8 ± 0.9 ^c^	79.8 ± 2.4 ^a^	747.3 ± 15.7 ^a^	859.1 ± 18.9 ^a^	228.8 ± 11.9 ^a^	388.6 ± 14 ^a^	340.7 ± 4.2 ^b^
*Q. rotundifolia*	69.2 ± 0.4 ^a^	82.6 ± 0.1 ^a^	482.5 ± 6.6 ^d^	681.3 ± 3.3 ^bc^	60.9 ± 5.2 ^c^	264.7 ± 8 ^c^	533.5 ± 5.7 ^a^

Values are mean ± SD. Different letters in the same column indicate statistically significant differences between acorn flour samples according to Tukey HSD test (*p* < 0.05). All evaluated samples correspond exclusively to the acorn flour (kernel) fraction. *Q. pubescens*, *Q. ilex*, *Q. cerris*, *Q. petraea*, and *Q. robur* (2024) originate from Croatia, whereas *Q. rotundifolia* (2024) originates from Portugal. *Q. suber* was excluded from these analyses due to flour biomass constraints.

## Data Availability

The original contributions presented in this study are included in the article/Supplementary Materials. Further inquiries can be directed to the corresponding authors.

## Supplementary Materials

The following supporting information can be downloaded at https://www.mdpi.com/article/10.3390/foods15193398/s1, Table S1: Pasting properties of acorn flours.

## Abbreviations

The following abbreviations are used in this manuscript:

AgNO_3_	Silver nitrate
ALA	α-linolenic acid
AM	Amylose
ANOVA	Analysis of variance
AOAC	Association of Official Analytical Chemists
BU	Brabender Units
CHO	Available Carbohydrates
dw	Dry weight
FA	Fatty acids
FN	Falling number
fw	Fresh weight
GC	Gas chromatography
MUFA	Monounsaturated fatty acids
MVA	Micro-visco-amylograph
n.d.	No data
OAC	Oil absorption capacity
PUFA	Polyunsaturated fatty acids
SFA	Saturated fatty acids
TDF	Total dietary fiber
TFA	Total fatty acids
TS	Total starch
WAC	Water absorption capacity
