# Supplementary material for: Comparative Nutritional, Rheological, and Lipid Profiling of Acorn Flours from Seven Quercus Species: A Scientific Framework for Functional Food Applications
Source: Foods. 2026 Sep 24;15(19):3398. doi: 10.3390/foods15193398 (PMC13664267; doi:10.3390/foods15193398)
Supplement: Supplementary file 1 [file foods-15-03398-s001.zip › foods-4516884-supplementary.pdf]

Table S1. Pasting properties of acorn flours.

Species	Pasting temperature (°C)	Peak temperature (°C)	Peak viscosity (BU)	Final viscosity (BU)	Breakdown viscosity (BU)	Setback viscosity (BU)	Falling number (s)
Q. cerris (H_2O)	67.1±0.5^{c}	80.3±3.9^{ab}	362.6±1.9^{f}	447.6±4.4^{f}	56.0±3.9^{d}	148.9±8.1^{e}	87.9±1.4^{d}
Q. cerris ($AgNO_3$)	66.5±1.0^{c}	81.0±0.4^{a}	379.6±4.6^{f}	455.5±3.9^{f}	57.9±6.6^{d}	143.9±11.6^{e}	97.9±5.7^{d}
Q. ilex (H_2O)	60.1±0.2^{d}	72.8±2.9^{d}	687.4±13.2^{b}	825.2±13.5^{b}	174.9±11.3^{b}	328.7±9.3^{b}	336.1±21.9^{b}
Q. ilex ($AgNO_3$)	59.5±0.6^{de}	73.8±1.8^{bcd}	696.3±17.3^{b}	839.2±14.2^{ab}	175.8±15.9^{b}	335.7±11.9^{b}	346.2±21.9^{b}
Q. petraea (H_2O)	68.2±0.1^{bc}	82.6±0.4^{a}	551.4±3.0^{d}	728.3±5.9^{c}	84.9±2.5^{c}	270.7±4.6^{c}	334.6±11.3^{b}
Q. petraea ($AgNO_3$)	68.0±0.4^{bc}	83.2±0.9^{a}	559.4±7.4^{d}	716.3±11.3^{c}	96.9±3.3^{c}	271.7±5.6^{c}	349.2±16.3^{b}
Q. robur (H_2O)	69.9±0.4^{ab}	80.1±0.2^{ab}	587.4±4.5^{c}	640.3±5.8^{e}	190.8±9.6^{b}	252.7±1.8^{cd}	150.9±0.1^{c}
Q. robur ($AgNO_3$)	70.1±0.2^{a}	78.8±0.5^{abcd}	598.4±9.1^{c}	637.4±7.4^{e}	195.8±13.1^{ab}	244.8±6.6^{d}	160.8±19.8^{c}
Q. pubescens (H_2O)	58.6±0.3^{de}	73.1±0.8^{cd}	738.4±11.1^{a}	843.3±8.0^{ab}	216.8±14.2^{ab}	377.7±9.4^{a}	339.2±2.1^{b}
Q. pubescens ($AgNO_3$)	57.8±0.9^{e}	79.8±2.4^{abc}	747.3±15.7^{a}	859.1±18.9^{a}	228.8±11.9^{a}	388.6±14^{a}	340.7±4.2^{b}
Q. rotundifolia (H_2O)	70.1±0.1^{a}	82.4±1.1^{a}	472.6±6.0^{e}	683.3±7.4^{d}	58.9±3.1^{d}	268.7±10.2^{c}	506.0±24.7^{a}
Q. rotundifolia ($AgNO_3$)	69.2±0.4^{ab}	82.6±0.1^{a}	482.5±6.6^{e}	681.3±3.3^{d}	60.9±5.2^{d}	264.7±8.0^{c}	533.5±5.7^{a}

*Values are mean ± SD. Different letters in the same column indicate statistically significant difference between acorn flour samples according to Tukey HSD test ($p < 0.05$).
